# Comparison of low molecular weight heparin, aspirin, and their combination for the prevention of thrombosis after total knee arthroplasty in obese patients

**DOI:** 10.1002/jeo2.70218

**Published:** 2025-03-18

**Authors:** Alireza Mirahmadi, Pooya Hosseini‐Monfared, Shahrzad Ghane, Mohammad Mortazavi, Ramin Abrishami, Mohammad Hossein Hooshangi, Vahid Shameli, Seyed Morteza Kazemi

**Affiliations:** ^1^ Bone Joint and Related Tissues Research Center Shahid Beheshti University of Medical Sciences Tehran Iran; ^2^ Clinical Department Pharmacy school, Islamic Azad University Tehran Medical Sciences Tehran Iran

**Keywords:** anticoagulant, aspirin, Clexane, deep venous thrombosis, obesity, total knee arthroplasty

## Abstract

**Purpose:**

Patients undergoing total knee arthroplasty (TKA) are at a high risk of thromboembolic events, which is higher in obese patients. Determining the appropriate prophylaxis for venous thromboembolism (VTE) in obese patients is challenging. Therefore, we aimed to compare the effects of low molecular weight heparin (LMWH) with aspirin (ASA) and their combination for the prevention of thromboembolic events after TKA in obese patients.

**Methods:**

In a retrospective study, 245 obese patients with BMIs over 30 who underwent TKA were enroled. Eligible patients were divided into three groups: Group A was given LMWH sodium (Clexane®) for 14 days, Group B was given ASA for 14 days, and Group C was given LMWH sodium (Clexane®) for 5 days and then ASA twice daily for the days between 5 and 14 postoperatively. The primary outcome was the incidence of VTE within three months. Secondary outcomes included routine laboratory evaluations (PT, PTT, INR, Hb, Hct, platelets, BUN and Cr) and adverse effects of ASA and LMWH, such as bleeding, anaemia, thrombocytopenia, and gastrointestinal or neurological symptoms.

**Results:**

Regarding the incidence of DVT and PTE, we did not observe significant differences between groups (*p* > 0.05). A total of seven symptomatic VTE was observed in six patients. We observed two cases with PE who were in the Clexane group. Moreover, five individuals had DVT in the follow‐up: three cases in the Clexane group, one in the ASA group, and one in the ASA + Clexane group, which was not statistically significant (*p* > 0.05). There were no differences between groups regarding the risk of adverse events and complications.

**Conclusion:**

We found that ASA is not inferior to enoxaparin in reducing VTE after TKA in obese patients. Therefore, given ASA's low cost and greater convenience, it may be considered a reasonable alternative for extended VTE prophylaxis for TKA surgery in obese patients.

**Level of Evidence:**

Level III.

**Abbreviations**
AAOS: American Academy of Orthopaedic Surgeons
ASA: aspirin
BMI: body mass index
BUN: blood urea nitrogen
Cr: creatinine
DOAC: direct‐acting oral anticoagulants
DVT: deep venous thrombosis
ESR: erythrocyte sedimentation rate
GI: gastrointestinal
Hb: haemoglobin
Hct: haematocrit
INR: international normalised ratio
LMWH: low molecular weight heparin
MCID: minimal clinically important difference
PE: pulmonary embolism
PJI: periprosthetic joint infection
PT: prothrombin time
PTT: partial thromboplastin time
TJA: total joint arthroplasty
TKA: total knee arthroplasty
VTE: venous thromboembolism

## INTRODUCTION

Every year, about 2.5 million total knee arthroplasty (TKA) surgeries are performed worldwide and help patients gain proper therapeutic outcomes such as pain reduction, improvement of ability, mobility, and quality of life [28]. Similar to other surgeries, venous thromboembolism (VTE) is among the main complications that have been studied to be prevented in TKA [33, 40]. Symptomatic deep venous thrombosis (DVT) is present in 0.5%–4.0% of patients after total hip or knee replacement, with 0.2%–1.9% leading to acute PE, even after adequate anticoagulant treatments [4, 26].

The obesity (body mass index (BMI) ≥ 30 kg/m^2^) rate has been increasing over the last two decades from 30.5% to 42.4% in the United States [20]. Obesity remains a serious health issue because of its potential risk factor for several comorbidities, including hip and knee osteoarthritis, which require total joint arthroplasty (TJA) sooner than the normal population [5, 21, 35]. Recent studies indicate a coherent increase in the obesity rate and the need for primary and revision TJA in the past decade [17, 27, 28, 46, 47]. Obesity promotes the inflammatory and thrombotic state, and by increasing the possibility of VTE incidents two to six‐fold, it is considered to be an important risk factor for VTE [8, 65]. An accurate anticoagulant therapy after TJA is of great importance in obese patients who are more prone to VTE [53, 70]. Obese patients may also demonstrate altered pharmacokinetic and pharmacodynamic profiles compared to people of normal BMI, such as increased volume of distribution and increased renal clearance rates, possibly leading to lower peak plasma levels and shorter half‐lives of certain antithrombotic medications used for VTE prophylaxis, resulting in the need for a more careful dose modification in obese patients [50].

Anticoagulants are routinely recommended to be prescribed prophylactically after major orthopaedic surgery to prevent VTE, which can reduce the risk of thromboembolic events by approximately 50%–80% [6]. Given the patient's safety and the economic burden inflicted upon the healthcare system, post and perioperative guidelines are needed to improve VTE prevention. To the date of writing this article, there is no universal agreement regarding the most suitable prophylactic regimen for the prevention of VTE after TKA in morbidly obese patients [10, 44]. The American Academy of Orthopaedic Surgeons (AAOS), although recommending prophylaxis for VTE after TJA, does not clarify the medication type, dosage, and duration of treatment [42]. Other organisations, including the American College of Chest Physicians and American Society of Hematology, differ in suggesting medications such as aspirin (ASA), low molecular weight heparin (LMWH) such as enoxaparin, vitamin K antagonists (e.g., warfarin), an indirect inhibitor of factor Xa, and direct‐acting oral anticoagulants (DOACs), and other medications with different dosages and duration [1, 11, 29, 31]. It should be considered that choosing anticoagulants affects the risk of major bleeding and complications or unacceptable outcomes [9, 11, 61].

As stated earlier, a universal consensus is yet to be made regarding VTE prophylaxis after TJA, though aspirin twice daily (ASA BID) has been proven to be a promising and safe option in primary TJA [12, 13, 30]. ASA also has a lower cost and fewer complications than other drugs used for VTE prevention and has proven to be similar in terms of efficacy compared to warfarin, LMWHs, and Xa inhibitors [3, 15, 24, 49, 55, 56, 63]. The safety of ASA compared to other VTE prophylaxis medications contributes to less morbid complications such as wound complications, periprosthetic joint infection (PJI), gastrointestinal (GI) bleeding, and mortality [48, 49, 58]. LMWH is another safe agent used in TKA that has good bioavailability and a long half‐life [23]. It is administered once daily subcutaneously without the need for laboratory monitoring [22]. LMWH (e.g., enoxaparin) has been shown to be an effective agent for VTE prophylaxis after TKA [62]. However, some disadvantages have been reported for LMWH, including potential thrombocytopenia, poor patient adherence, parenteral administration and expenditure [69, 71].

As mentioned, obesity has increased the need for TKA and, as a prothrombotic state, has increased the incidence of VTE after TKA. Current guidelines lack exclusive solutions to VTE prevention in obese patients due to the lack of proper clinical studies explicitly examining this population [68]. Moreover, the International Society on Thrombosis and Haemostasis's (ISTH) Scientific and Standardization Committee recommends against the use of DOACs in BMI > 40 kg/m^2^ or weight > 120 kg and drug levels checking because of the issue mentioned earlier [34]. To the best of our knowledge, there is not enough study about the efficacy of LMWH as compared to ASA on the occurrence of VTE in obese patients. In non‐obese patients who underwent TKA, the ASA has shown a promising result with lower side effects compared to LMWH; [14] therefore, this study was designed to evaluate and compare the laboratory parameters, complications, and VTE occurrence after TKA in obese patients receiving Enoxaparin (Clexane™), ASA, and those who received both LMWH and ASA.

## METHODS AND MATERIALS

### Participants

This retrospective study was conducted in an orthopaedic referral hospital from 2014 to 2023. Inclusion criteria consisted of patients referred to the orthopaedic department with knee osteoarthritis and underwent primary unilateral TKA, body mass index (BMI) > 30 kg/m^2^, without a current or previous history of VTE. Exclusion criteria consisted of patients who underwent revision TKA, patients with septic arthritis, inflammatory arthritis such as rheumatoid arthritis [54], patients with peripheral artery disease, varicose veins, malignancy, thrombocytopenia (platelet count less than 1.2 × 10^5^/mm^3^), anaemia (Hb < 11 g/dL), primary and secondary haemostasis disorders or bleeding tendency during the preoperative coagulation test, history of liver or kidney disease, history of aspirin or heparin allergy receiving anticoagulant drugs 15 days before surgery [19], and age below 35 years or more than 85 years. We also excluded patients with incomplete data. The study was performed according to the declaration of Helsinki and was approved by the Ethics Committee of Shahid Beheshti University of Medical Sciences (IR.SBMU.REC.1397.035).

### Study design and surgery

The three groups in our study each received a different postoperative anticoagulant treatment. Group A was given LMWH sodium (Clexane®; Sanofi Corp, Paris, France) 4000 UI (40 mg) once a day for 14 days, Group B was given ASA (Poursina Corp, Tehran, Iran) 80 mg twice daily for 14 days starting 12 h after the surgery, and Group C received LMWH 40 mg once daily for 5 days and then received ASA for the other days up to day 14 postoperatively [18, 45]. Anaesthesia methods have been recorded and compared between the groups. Spinal anaesthesia was delivered using 0.5% ropivacaine (Molteni & C Dei Fratelli, Italy) in combination with fentanyl (25–50 µg). For intraoperative sedation, an infusion of 2% propofol at a rate of 7–10 mL/h (Fresenius Kabi Austria GmbH, Austria) was used, with dosage adjustments made at the anesthesiologist's discretion. All TKAs were performed by the same surgeon using the standard medial parapatellar approach following an anterior midline skin incision. The cemented Scorpio NRG Knee System (Stryker, Kalamazoo, Michigan, USA) without patellar resurfacing was utilised. The tourniquet with a pressure of 100 mmHg above systolic blood pressure was used from the skin incision to the end of cementing for all the patients. A single dose of tranexamic acid (1 g) was used intraarticularly for all the patients.

The patients were dressed with an elastic bandage and a knee immobilizer after the TKA during the hospitalisation period. Patients underwent a similar postoperative pain control and rehabilitation protocol, which included walking exercises and strength training for lower extremity muscles, beginning on the first postoperative day. Also, patients were encouraged to walk as much as possible with supervision from nursing staff or their families.

### Measurements and follow‐ups

The primary outcome measure of this study was the incidence of VTE events such as DVT and PE. At the postoperative stage, any evidence of DVT and PE, including oedema, swelling in the leg, pain in the calf or thigh, warmth and redness of the leg, chest pain, short breath, tachypnea, and tachycardia was recorded by a single orthopaedic surgeon. In the third month after surgery, a Doppler ultrasound was performed on the deep veins of both lower extremities, and the possibility of thrombosis of the common and superficial femoral, popliteal, posterior tibial, and muscular calf veins was evaluated by a single radiologist.

Secondary outcomes assessed included alterations in laboratory parameters and the incidence of adverse events associated with thromboprophylaxis agents. The patients underwent routine examinations and lab workups at the preoperative stage and on the last day of hospitalisation (postoperative Day 3), including routine anticoagulation measures (prothrombin time [PT], partial thromboplastin time [PTT], and INR), blood tests (haemoglobin (Hb), haematocrit (Hct), platelet, blood urea nitrogen (BUN) and creatinine (Cr). Patients were also evaluated for the common adverse effects of ASA or LMWH, including bleeding complications, anaemia, thrombocytopenia, heartburn, diarrhoea, nausea, headache, and drowsiness. We also recorded the patients' demographic and surgical data, including age, gender, BMI and anaesthesia type. All of the patients were followed for 3 months.

### Data analysis

Statistical analysis was performed using SPSS statistical software version 29 (SPSS Inc., Chicago, IL, USA). The Kolmogorov–Smirnov test was used to check the normality of the variables. One‐way analysis of variance and students' t‐test were used to analyse normally distributed continuous variables. In addition, we used the Kruskal–Wallis and Mann–Whitney *U* test to analyse non‐normally distributed continuous variables. Paired T‐test and Wilcoxon's tests were used for paired analysis in the parametric and nonparametric variables, respectively. The Pearson Chi‐Square test was used for categorical variables analysis, and Spearman and Pearson's correlation tests assessed associations between the variables. *p*‐Values less than 0.05 were considered statistically significant.

## RESULTS

**Figure 1:**
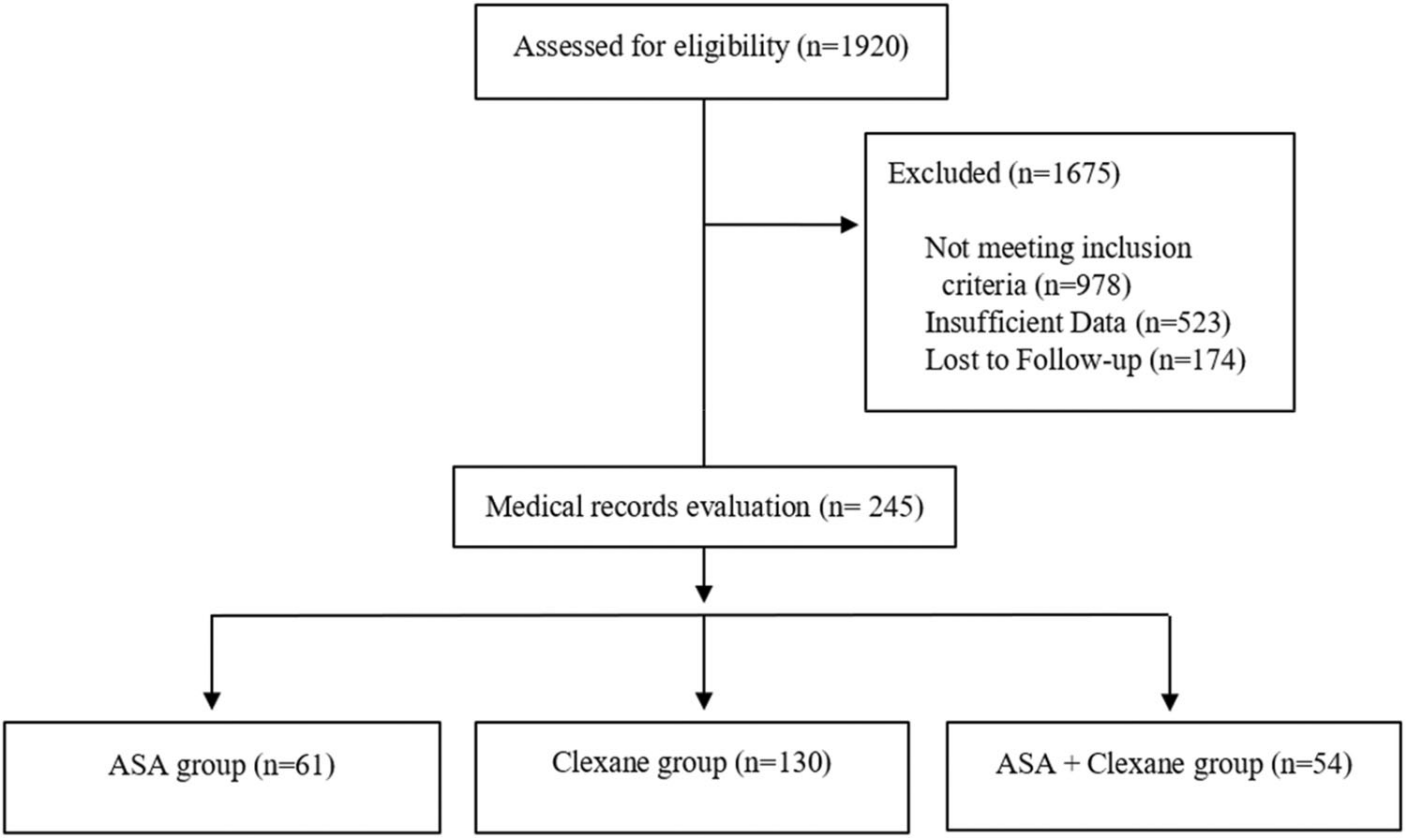
Study flow diagram. ASA, aspirin.

In the beginning, 1920 patients diagnosed with knee osteoarthritis needing TKA, who had been diagnosed by an orthopaedic surgeon based on clinical and para‐clinical findings, were evaluated regarding inclusion and exclusion criteria. Finally, 245 patients were included in the study; 130 were in the Clexane group, 61 in the ASA group, and 54 in the ASA + Clexane group (Figure 1).

The included patients had a mean age of 66.91 (56–78) and a mean BMI of 35.12 ± 3.17. Demographic features in terms of age (*p* = 0.227), sex (*p* = 0.440), BMI (*p* = 0.435) and other studied variables such as the side of TKA (*p* = 0.575) and type of anaesthesia (*p* = 0.372) were similar between groups (Table 1). The preoperative variables, including laboratory findings, did not show significant differences between the groups (*p* > 0.05) (Table 2).

**Table 1:** Characteristics of the patients in the three groups.

**Variables**	**ASA (*n*** = **61)**	**Clexane (*n*** = **130)**	**ASA + Clexane (*n*** = **54)**	***p*‐Value**
Gender				
Male	13 (21.3)	20 (15.4)	7 (13.0)	0.440
Female	48 (78.7)	110 (84.6)	47 (87.0)	
Age (year)	68.41 ± 7.94	66.34 ± 9.01	66.59 ± 6.66	0.227
BMI	34.31 ± 2.92	35.10 ± 3.09	35.94 ± 3.49	0.435
Side of TKA				
Right	31 (50.8)	58 (44.6)	28 (51.9)	0.575
Left	30 (49.2)	72 (55.4)	26 (48.1)	
Anaesthesia				
Spinal	52 (85.2)	114 (87.7)	43 (79.6)	0.372
General	9 (14.8)	16 (12.3)	11 (20.4)	

*Notes*: Mean ± SD and *N* (%).

Abbreviations: ASA, aspirin; BMI, body mass index; SD, standard deviation; TKA, total knee arthroplasty.

**Table 2:** Preoperative laboratory values.

Variables	ASA (*n* = 61)	Clexane (*n* = 130)	ASA + Clexane (*n* = 54)	*p*‐Value
Haematocrit	39.54 ± 4.17	38.58 ± 3.83	39.10 ± 3.52	0.522
Haemoglobin (g/dL)	12.97 ± 1.60	12.68 ± 1.47	12.76 ± 1.29	0.624
Platelet (10^9^/L)	261 ± 62.73	262 ± 67.80	237.61 ± 64.85	0.096
BUN (mg/dL)	37.37 ± 12.77	40.09 ± 12.48	36.81 ± 8.74	0.097
Cr (mg/dL)	0.91 ± 0.24	1.03 ± 0.68	0.91 ± 0.17	0.166
ESR (mm/h)	19.76 ± 16.31	23.25 ± 18.74	21.97 ± 12.49	0.297
PT (s)	11.96 ± 0.69	12.62 ± 0.93	12.62 ± 0.54	**0.000** ^*^
PTT (s)	32.95 ± 4.54	31.51 ± 4.91	32.06 ± 4.04	0.160
INR	1.04 ± 0.09	1.05 ± 0.08	1.02 ± 0.05	0.098

*Note*: *Significant at 0.05 level.

Abbreviations: ASA, aspirin; BUN, blood urea nitrogen; Cr, creatinine; ESR, erythrocyte sedimentation rate; INR, international normalised ratio; PT, prothrombin time; PTT, partial thromboplastin time.

Results showed that serum levels of haematocrit, haemoglobin and platelet decreased significantly in groups as compared to serum levels before intervention (*p* < 0.05). At the same time, BUN in the Clexane group decreased significantly after intervention (*p* = 0.027), which remains non‐significant in the ASA and also the ASA + Clexane group (*p* = 0.972). Moreover, the erythrocyte sedimentation rate (ESR) increased in all the groups after TKA. Analysis between groups showed that the rise of the ESR in the Clexane group was more prominent in comparison to the ASA group (*p* < 0.05) (Table 3).

**Table 3:** Postoperative laboratory values, mean ± SD.

Variables	ASA (*n* = 61)	Clexane (*n* = 130)	ASA + Clexane (*n* = 54)	*p*‐Value
Haematocrit	33.3 ± 4.56*	31.91 ± 3.93*	32.77 ± 3.56*	0.229
Haemoglobin (g/dL)	11.02 ± 1.61*	10.73 ± 1.95*	10.79 ± 1.29*	0.423
Platelet (10^9^/L)	219 ± 54.93*	232 ± 65.1*	207.92 ± 56.08*	0.148
BUN (mg/dL)	42.13 ± 19.51	37.43 ± 13.69*	38.33 ± 9.57	0.656
Cr (mg/dL)	0.94 ± 0.26	1.04 ± 0.30	0.94 ± 0.15	0.284
ESR (mm/h)	27.12 ± 16.19*	58.53 ± 37.59*	41.45 ± 31.25*	0.136
PT (s)	12.73 ± 0.66	13.2 ± 0.7	13.14 ± 0.57*	0.352
PTT (s)	36.62 ± 3.02	33 ± 5.36	32.98 ± 4.53	**0.009** **
INR	1.06 ± 0.07	1.05 ± 0.09	1.06 ± 0.08*	0.741

Abbreviations: ASA, aspirin; BUN, blood urea nitrogen; Cr, creatinine; ESR, erythrocyte sedimentation rate; INR, international normalised ratio; PT, prothrombin time; PTT, partial thromboplastin time; SD, standard deviation.

* Variables that demonstrated significant change after the surgery compared to their value at preoperative evaluations.

** Significant at 0.05 level.

Comparing the incidence of DVT and PE in groups, we did not observe significant differences between groups (*p* > 0.05). A total of seven symptomatic VTE was observed in six patients (one case had both DVT and PE in the Clexane group). We observed two cases with PE after three months of follow‐up, who were in the Clexane group. Moreover, five individuals had DVT in follow‐up: three cases in the Clexane group, one case in the ASA group, and one case in the ASA + clexane group, which was not statistically significant (*p* > 0.05). All six patients in the groups had symptomatic VTE, and we did not observe asymptomatic VTE in regular Doppler ultrasound of the deep veins of the lower extremities. Based on previous studies, minimal clinically important difference (MCID) change for different anticoagulant agents was considered 2% regarding the thrombosis rate [43]. As shown in this study, the differences between the incidence of thromboembolic events were less than 2% between groups, which shows that the differences between these anticoagulant agents do not reach the desired MCID.

No complications, such as intracranial haemorrhaging or massive haemorrhaging of the GI tract, were found in any of the groups. There were no differences in the risk of adverse events, such as bleeding, wound complications, myocardial infarction, and death, when aspirin was compared with LMWH (Table 4).

**Table 4:** Incidence of thromboembolic events and related symptoms.

Variables	ASA (*n* = 61)	Clexane (*n* = 130)	ASA + Clexane (*n* = 54)	*p*‐Value
Chest pain	0	2 (1.5)	0	1
Shortness of breath	0	2 (1.5)	0	1
Tachypnea	0	2 (1.5)	0	1
Tachycardia	0	2 (1.5)	0	1
PE occurrence after 3 months	0	2 (1.5)	0	1
Oedema	26 (42.6)	48 (36.9)	18 (33.3)	0.577
Pain in the calf or thigh	30 (49.2)	51 (39.2)	24 (44.4)	0.417
Warmth and redness	9 (14.8)	14 (10.8)	8 (14.8)	0.641
DVT after 3 months	1 (1.6)	3 (2.3)	1 (1.9)	1
Fever	6 (9.8)	7 (5.4)	5 (9.3)	0.416
Infectious	3 (4.9)	7 (5.4)	5 (9.3)	0.526
Bleeding	4 (6.6)	10 (7.7)	4 (7.4)	1
Total VTE occurrence	1 (1.6)	4 (3.1)	1(1.9)	1

Abbreviations: ASA, aspirin; DVT, deep vein thrombosis; PE, pulmonary embolism; VTE, venous thromboembolism.

## DISCUSSION

The findings of our study demonstrated that the prophylactic use of ASA compared to LMWH and a combination of ASA and LMWH was not associated with a significant difference in the incidence of thromboembolic events such as PE or DVT in obese patients who underwent TKA. Also, the complications associated with the use of anticoagulants, such as bleeding, were not different between the three groups, indicating a similar safety profile of these two drugs in obese patients who underwent TKA.

A recent meta‐analysis of six studies by Meng et al. [38] investigating the effect of aspirin against LMWH for VTE prophylaxis after TKA demonstrated that the rate of DVT was not significantly different between the two groups. However, the overall VTE rate was lower in the LMWH group. The evaluated studies in this meta‐analysis included patients who underwent both total knee and total hip arthroplasties that have different VTE incidence rates as Humphrey et al. found that morbidly obese patients who underwent TKA are at a four times higher risk of developing VTE compared to morbidly obese patients who underwent THA [25]. Also, some of the included studies had variability in their interventions, as an included study compared the ASA plus VenaFlow with LMWH plus VenaFlow or studies that used different tranexamic regimens alongside these agents. Another systematic review and meta‐analysis conducted by Matharu et al. [36] in 2020 included 13 RCTs comparing the different anticoagulant agents such as ASA, warfarin, rivaroxaban, LMW dextran, Dipyridamole, and LMWH. They demonstrated that the incidence of VTE events and bleeding complications were not significantly different between ASA and other anticoagulants. The antithrombotic intervention types and dosage, including both mechanical and chemical methods, used in this meta‐analysis's included studies displayed high heterogeneity. Also, the studies included in this meta‐analysis have included data on both total hip and knee arthroplasties. However, it is not appropriate to generalise the findings of this study to knee arthroplasty, as patients undergoing knee arthroplasty have a higher rate of VTE events compared to hip arthroplasty. Additionally, VTE tends to manifest earlier following knee arthroplasty [41, 51, 57].

In a study by Sidhu et al. [59] that included 9711 patients who underwent either hip or knee arthroplasty, it was observed that patients who received aspirin had a higher rate of symptomatic VTE within 90 days after the arthroplasty. However, the incidence rate of pulmonary embolism and above‐knee DVT was not different in the two groups. Also, bleeding complications and mortality were not different in patients who received aspirin compared to those who received LMWH [59]. Our findings aligned with this study's findings regarding pulmonary embolism and bleeding complications. However, we did not observe any significant difference in the incidence of any VTE events between the two groups. Our study population included only knee arthroplasties, in contrast to their study, which included both hip and knee arthroplasties. Furthermore, our study population consisted of patients with BMIs over 30 who had a higher risk of thromboembolic events, but the mentioned study did not consider BMI categorisation as an important factor in doing a subgroup analysis in obese patients. Few previous studies have investigated the efficacy of different antithrombotic agents in this high‐risk population. Our findings demonstrated that although obesity puts these patients at higher risk of complications and VTE following TKA, aspirin has the same efficacy as LMWH for thromboembolic prophylaxis.

In a retrospective study of patients who underwent elective lower limb arthroplasty, Sloan et al. evaluated the incidence rate of VTE and PE according to different BMI groups [60]. Their findings indicated that patients who are classified as overweight and obese based on their BMI are at higher risk of developing PE, but the risk of DVT development was not different between the groups [60]. Pineo et al. [52] compared the efficacy of apixaban and enoxaparin in different subgroups and found that the rate of VTE events is not different between the two groups at different BMI subgroups. A recent study comparing the rate of thromboembolic complications in morbid obese (BMI > 40) patients receiving either ASA or other anticoagulants following TJA demonstrates that ASA is a safe anticoagulant option in this high‐risk group [64].

The need for dosage adaptation for VTE prophylaxis in obese patients by LMWH was investigated by Vavken et al. [67]. They compared the high‐dose (5000 IUs) and low‐dose (3500 IUs) LMWH in 723 obese patients who underwent orthopaedic surgeries and found that the high‐dose LMWH did not significantly reduce the incidence of postoperative VTE compared to the low‐dose LMWH [67]. In this study, we utilised the standard dose of enoxaparin of 4000 IU daily for the LMWH group.

In a study by Melinte et al. [37], it was shown that high inflammatory markers in the blood samples of patients can predict the incidence of acute DVT following TKA. ESR was increased in all the groups, and the increase in the LMWH and ASA + LMWH groups was significantly higher than the ASA group, which may be due to the anti‐inflammatory effects of ASA [66]. If pre and postoperative inflammatory markers such as ESR are considered predictors of VTE events, maybe ASA could reduce the rate of VTE in obese patients more than LMWH [16, 72, 73]. However, the rate of VTE events is not statistically different in our study, and prospective studies with larger sample sizes are required to investigate this correlation.

Also, in our study, BUN significantly changed after the TKA in the LMWH groups. BUN was lower in patients in the LMWH group and ASA + LMWH compared to the ASA group. As LMWH did not increase post‐operation BUN similar to other groups, maybe it could be considered a better choice in patients with renal impairment. A study on patients older than 75 with moderate renal impairment investigated the use of dabigatran compared to enoxaparin and found that enoxaparin is as effective as dabigatran as a thromboprophylaxis agent [7]. Furthermore, elevated dehydration indicated by a BUN/Cr ratio greater than 20 has been proposed as a potential risk factor for VTE events after TKA [32]. Given that patients with renal impairment are more susceptible to dehydration [2], the lower BUN/Cr ratio observed in the LMWH group in the study results underscores the importance of conducting additional research to assess the effectiveness of LMWH in preventing DVT in patients with renal impairment.

Our study had some limitations. First, due to the low prevalence of obese patients, the sample size was limited, and the results might not represent the real‐world population of obese patients. Second, this study's retrospective and observational nature might result in not controlling other possible influential variables. We advise further prospective controlled clinical trials on obese patients undergoing joint arthroplasty with a larger sample size to increase the depth of our knowledge in anticoagulation therapy for obese patients. The tourniquet was used from incision to cementation with the purpose of shorter operative time, better visibility, reduced blood loss, and dry bone surface for better cement interdigitation, as well as implant survivorship in this study for all the patients, which may affect the rate of VTE rates compared to studies that did not use the tourniquet [39]. It would also be interesting to explore the possibility of using ESR as a prognostic factor for VTE in patients receiving anticoagulant therapy. Since enoxaparin, unlike other groups, did not increase BUN, the use of this drug in patients with renal impairments could also be considered a subject for future studies.

## CONCLUSIONS

This study showed that ASA is not inferior to LMWH in reducing VTE after TKA in obese patients. Therefore, given ASA's low cost and greater convenience, it may be considered a reasonable alternative for VTE prophylaxis after TKA in obese patients. Additional high‐quality clinical trials are needed to provide definitive conclusions regarding the comparative clinical effectiveness of ASA versus LMWH in reducing VTE incidence after TKA in obese patients.

## AUTHOR CONTRIBUTIONS

All authors contributed to the study conception and design. Alireza Mirahmadi, Pooya Hosseini‐Monfared, Mohammad Mortazavi, Vahid Shameli, and Shahrzad Ghane participated in data gathering. Alireza Mirahmadi and Pooya Hosseini‐Monfared performed data analysis. Alireza Mirahmadi, Pooya Hosseini‐Monfared, Mohammad Mortazavi, Ramin Abrishami, and Shahrzad Ghane drafted the manuscript. Vahid Shameli, Mohammad Hossein Hooshangi, and Seyed Morteza Kazemi revised the manuscript. All authors read and approved the final version of the manuscript.

## CONFLICT OF INTEREST STATEMENT

The authors declare no conflict of interest.

## ETHICS STATEMENT

This study was performed in line with the principles of the Declaration of Helsinki. Approval was granted by the Ethics Committee of Shahid Beheshti University of Medical Sciences (IR.SBMU.REC.1397.035).

## Data Availability

The data sets used and/or analysed during the current study are available from the corresponding author on reasonable request.
